# Restoring Partial Loss of the Teeth without Plate

**Published:** 1881-07

**Authors:** 


### RESTORING PARTIAL LOSS OF THE TEETH WITHOUT PLATE.

BY LOW'S NEW METHOD.

By this method the ordinary mechanical laboratory tools are dispensed with, in a measure. No impression is needed except for the selection of the teeth, consequently no care need be taken as to its perfection.

This method consists in attaching permanently to the adjacent teeth artificial teeth by means of watertight immovable perfectly fitting gold bands on either side of the space to be supplied, whence it receives its support. The ordinary unpleasant process of *plaster* casts, and metal dies, for swedging are thereby dispensed with.

Heretofore artificial teeth have invariably been supported entirely by the gums, and usually upon a plate held in its position by suction, or by clasp attachments; but by this method the presure in masticating is upon the teeth themselves. By the method here suggested spaces are left them for rinsing and cleaning, as with the natural teeth; so that the chance for an accumulation of filth is an utter impossibility more than around the natural teeth.

The use of the Plate for supporting the artificial teeth are higly objectionable because they cover mucous surfaces which health requires should be uncovered; secondly they occupy space within the mouth and are generally uncomfortable. They also require frequent removal for the purpose of cleansing, or they promote disease. The use of clasps to retain the teeth in position by using small supporting plates has very generally been abandoned on account of injury to the teeth clasped, caused by the continual friction of the band against the teeth, and the suction plate has been substituted. The clasp is supposed to be more objectionable than the discomfort and inconveniences attending the use of the suction plate. But time has shown, I believe, that even suction plates as they are generally used in close contact with the teeth, have destroyed more teeth than the clasp plate by causing recession of the gums, from being covered by vitiated stagnant secretions, held there even where the usual degree of cleanliness has been observed. Then again teeth partially covered as with the majority of partial plates made, that portions covered near the margin of the gums become so sensitive that many times the patient insists on having them removed. I will say however that I have never extracted any of these teeth, but have known of many teeth being sacrificed on that account. I mention a few of these facts to show that there are many serious objections in our present manner of supplying partial loss of teeth.

Where a person has been unfortunate enough to loose the full number of teeth, then they must resort to the next best thing be contented like a person who has been supplied with an artificial limb, which is better than nothing with all its objections.

Now in presenting this subject to the profession I am aware of the many objections that will be urged against its use, by those who have not had the opportunity of investigating its merits. Notwithstanding I claim to have surmounted and removed the chief difficulties that have proved so objectionable in supplying partial sets of teeth. These claims have not been hastily made for I have been giving this subject my attention for over five years and have now in actual service over six hundred cases, from one to five teeth in a span, dating back between five and six years down to the present time.

My manner of doing this work is as follows:

Pure block tin rolled into thin strips of about No. 34 in thickness or less if necessary, in order to pass between the teeth, is used to take the impression or form of the teeth adjacent to the space to be supplied; the tin is bent around the tooth and carefully pressed with flat pliers the length of the tooth, giving the perfect form of the tooth, this is taken off of the tooth and cut by the mark made by the pliers and used as a pattern from which a gold band is made. (The gold should be coin rolled out to 29 or 30 stubs gaged) and is soldered together with the following formula for solder: To one part gold add 4 grains coin silver and 2 grains of *pure* copper; this solder gives strength and a color not unlike the gold. These bands after being carefully soldered are fitted by being thriven slowly over the tooth with frequent annealings and cutting of points likely to come in contact with the gum until it is the shape of the process. Then cut the outer portion where it might show to a narrow band, leaving it full on the rest of the tooth until adjusted. Drive down to the gums but not under, cut off and bend over the crown of the tooth, where the articulation will permit (and there are very few cases where the entire portion of the crown is used in articulation). The bands being fitted, the teeth are arranged, being held in position by a compound of wax, gutta percha and rosin. Some strength is needed to retain the teeth until arranged; after they are all arranged to the satisfaction, a plaster impression is taken of the teeth and bands. The bands are taken off and placed in the impressions being particular to get them in proper position. Then pour with plaster mixed with asbestos: and when hard cut out on the lingual surface until the wax is reached; carefully remove the wax, back the teeth with gold and platina or clasp material and solder the bands and backings and finish, being careful not to bend your bands in polishing. I have found the attachment to be so rigid that extra strength was needed; but where there is a large span and greater strength needed, I make gold sockets, filling each tooth in its respective socket before arranging the teeth. The labial side I generally cut out a little, so as to show the tooth, if desired. The impression is taken as before described and the socket soldered together, after removing the teeth, thus avoiding the possibility of checking the teeth. The teeth can then, using the slot attachment, be soldered to perpendicular standing pins, be cemented in with any of the phosphates, making them very firm in their places. But very little danger need be apprehended from breakage, when used in this way: should such occur however the broken tooth can be removed and another be ground and fitted as before without removing the attachments. After the case is finished and ready for the mouth, great care must be taken to keep the teeth to be attached to, dry (to prevent moisture I use Gum Benzoin dissolved in alcohol on the gums some distance back of the teeth to be attached to). The cement is then mixed by the assistant, who coats the inside of the bands and the teeth belonging to said bands. Should it be difficult while operating to keep the moisture out, rubber dam may sometimes be used to advantage and afterwards pulled from under the attachment. The success of the operation in most cases depends on dryness, as much as in gold filling. The teeth are now nicely malleted to their places having previously been tried, to see that all was right. Should moisture begin to make its appearance, varnish over with the benzoine at once, immediately afterwards use water; thus preventing the alcohol from injuring the cement. After the cement is sufficiently hard, finish by beveling and burnishing all edges close to the teeth. If near the front of the mouth the small rim of the band outside can be made to exactly resemble the common marginal filling.

These attachments are immovable with *water tight* bands having no support on the gums and leaving no possibility of offensive accumulation any more than around the natural teeth and are as easily kept clean.—In this description I have been speaking of restoring molars and bicuspids of either superior or inferior maxilary. In restoring one, two, three or four front teeth, the ordinary plate teeth are used, resting the front of the tooth on the gum, leaving spaces between the teeth for cleansing. It is necessary to back the entire lingual surface of the teeth to prevent possible breakage during mastication, the gold only showing on the inside.

In addition to this there are many cases of elongated teeth which soon become loose and troublesome for want of use, where it would be impossible to use a plate of any kind. In such cases the same band attachment connecting with a truss or bar of gold is used with great satisfaction for masticating as well as for preserving the tooth so elongated.

The general supposition is that teeth so attached to, are injured, but experience has proved the contrary. I have yet to loose the first tooth, or discover any injury arising from such attachments either from loosening or decay. I have removed many bands after two or three years and some of four years standing; with the exception of the tooth being a little sensitive to heat and cold, no visible change had taken place.

